# Evolution after Introduction of a Novel Metabolic Pathway Consistently Leads to Restoration of Wild-Type Physiology

**DOI:** 10.1371/journal.pgen.1003427

**Published:** 2013-04-04

**Authors:** Sean Michael Carroll, Christopher J. Marx

**Affiliations:** 1 Department of Organismic and Evolutionary Biology, Harvard University, Cambridge, Massachusetts, United States of America; 2 Faculty of Arts and Sciences Center for Systems Biology, Harvard University, Cambridge, Massachusetts, United States of America; Université Joseph Fourier, France

## Abstract

Organisms cope with physiological stressors through acclimatizing mechanisms in the short-term and adaptive mechanisms over evolutionary timescales. During adaptation to an environmental or genetic perturbation, beneficial mutations can generate numerous physiological changes: some will be novel with respect to prior physiological states, while others might either restore acclimatizing responses to a wild-type state, reinforce them further, or leave them unchanged. We examined the interplay of acclimatizing and adaptive responses at the level of global gene expression in *Methylobacterium extorquens* AM1 engineered with a novel central metabolism. Replacing central metabolism with a distinct, foreign pathway resulted in much slower growth than wild-type. After 600 generations of adaptation, however, eight replicate populations founded from this engineered ancestor had improved up to 2.5-fold. A comparison of global gene expression in wild-type, engineered, and all eight evolved strains revealed that the vast majority of changes during physiological adaptation effectively restored acclimatizing processes to wild-type expression states. On average, 93% of expression perturbations from the engineered strain were restored, with 70% of these occurring in perfect parallel across all eight replicate populations. Novel changes were common but typically restricted to one or a few lineages, and reinforcing changes were quite rare. Despite this, cases in which expression was novel or reinforced in parallel were enriched for loci harboring beneficial mutations. One case of parallel, reinforced changes was the *pntAB* transhydrogenase that uses NADH to reduce NADP^+^ to NADPH. We show that PntAB activity was highly correlated with the restoration of NAD(H) and NADP(H) pools perturbed in the engineered strain to wild-type levels, and with improved growth. These results suggest that much of the evolved response to genetic perturbation was a consequence rather than a cause of adaptation and that physiology avoided “reinventing the wheel” by restoring acclimatizing processes to the pre-stressed state.

## Author Summary

Acclimatizing and adaptive (evolutionary) processes allow organisms to thrive despite cellular and environmental perturbations. Our work examined whether adaptation restores stress responses towards wild-type (pre-stressed) versus novel physiological states during adaptation by studying a bacterium (*Methylobacterium extorquens* AM1) that was experimentally engineered and evolved with a novel central metabolism. The engineered strain was much slower and less fit than wild-type, but eight replicate populations evolved for six hundred generations showed substantial improvements. We found that changes in gene expression during adaptation consistently restored acclimatizing processes to the wild-type state, often in 8/8 evolved lines. Novel changes were common and largely restricted to one lineage; however, highly parallel novel changes revealed loci harboring beneficial mutations. Even rarer were reinforced changes, such as *pntAB* transhydrogenase, which increased beyond immediate acclimation during evolution to restore NAD(P)(H) metabolism and improve growth. Overall, a few novel or reinforcing changes drove the mass-restoration of physiology back to wild-type.

## Introduction

Physiological stressors affect organisms across individual and evolutionary timescales: they invoke in individuals processes that work to restore homeostasis, and become over evolutionary timescales the selective pressures that drive adaptation in populations. How organisms generate innate and evolved responses to stressors – often termed physiological acclimation (or phenotypic plasticity) and adaptation, respectively – is a driving question today in many different fields of science, from the origins of drug resistance to the effects of global climate change. A common goal in many of these areas is to move from case-by-case studies towards a predictive understanding of how organisms will adapt to future stressors. However, whereas acclimatizing responses are generally “prewired” and relatively uniform between individuals of a population, the paths and outcomes of adaptation can be many and varied. Even under a simplified scenario of consistent selective pressures across replicate populations, evolution is not deterministic. There are many potential explanations for this variability - such as the randomness of mutations, escaping drift, epistasis, and clonal interference - all of which can give rise to multiple and sometimes quite disparate evolutionary outcomes [1]–[7]; yet, in other instances, adaptation is remarkably parallel between independently-evolved lineages, even down to the genetic level [8]–[10].

In replicate populations of laboratory-evolved organisms, parallelism is commonly interpreted as a sign of selection in either genetic [8] or phenotypic [11] data. Most studies determine the basis and parallelism of adaptation by comparing ancestral versus evolved states. However, in cases of adaptation to an environmental or genetic perturbation, there exists a third “wild-type” state that existed prior to the exposure to stressors that is often ignored. Exposure to genetic or environmental stressors invokes numerous processes that shift organisms from a wild-type to a perturbed physiological state, and it is this perturbed physiological state that is optimized over evolutionary timescales by natural selection. Thus, during experimental evolution all evolved strains share an initial set of acclimatizing responses that could be resolved differently by natural selection across replicate lineages. Given only a comparison of the ancestral (perturbed) and evolved states, it would be unclear how much of parallel adaptation represents convergent evolution of truly novel physiology, versus a wholesale restoration of cellular function to the pre-perturbed state. This has the potential to greatly conflate which physiological changes are likely causes versus consequences of improved fitness, and falsely identify highly parallel instances of adaptive evolution. To our knowledge, only one other study [12] has explicitly addressed the extent to which organisms adopt novel versus restored physiological states during adaptation to an environmental stressor, versus a genetic alteration.

**Figure 1:**
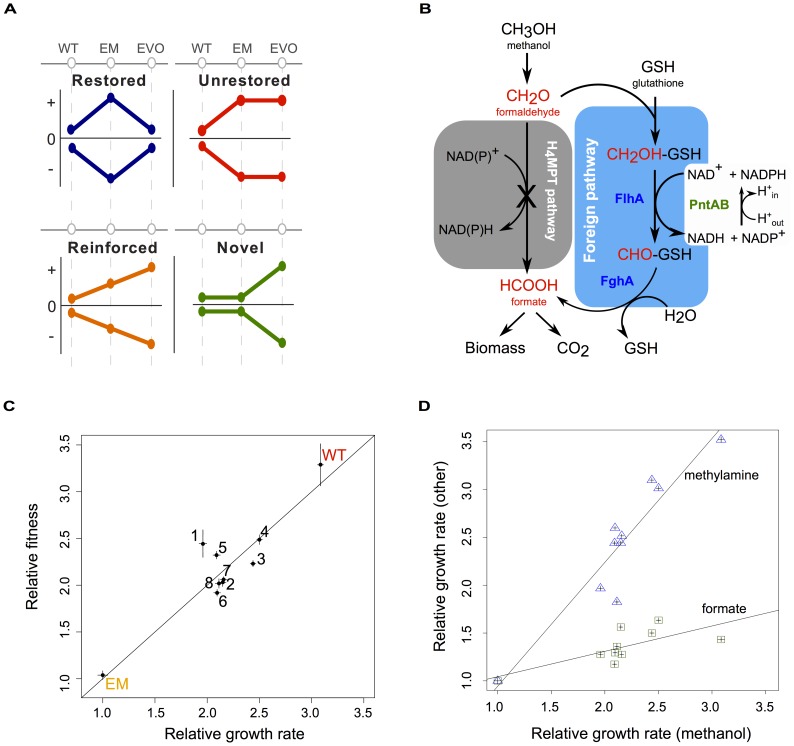
Acclimation and adaptation in an experimentally engineered and evolved bacterium. A) Combinations of acclimatizing and adaptive responses can be classified into four basic patterns based on wild-type (WT), perturbed (here, the engineered *Methylobacterium* strain, or “EM”), and evolved (EVO) physiological states. Physiological processes that were perturbed but return to a WT-like state are restored (blue); other processes that remain in a perturbed state are unrestored (red); those that are augmented from acclimation to adaptation are reinforced (orange); and still others are novel with respect to WT and EM states (green). B) Central one-carbon (C_1_) metabolism of WT and EM strains. In EM, the native pathway of formaldehyde oxidation (grey box) has been disabled and replaced by a foreign plasmid expressing two genes – *flhA* and *fghA*, from *Paracoccus denitrificans* – whose protein products co-opt endogenous glutathione to generate a functionally analogous, yet non-homologous substitute for C_1_ metabolism (blue box). This replacement results in the requirement for PntAB transhydrogenase to generate NADPH. C) EM was evolved in eight replicate cultures on methanol for over 600 generations. Isolates from each of the evolved populations (F1–F8) showed marked increases in growth rate and fitness relative to their EM ancestor. Line indicates y = x. D) Growth rates relative to EM on methanol are plotted for WT and the evolved isolates against two other C_1_ compounds: methylamine and formate. Lines show linear regression with an r^2^ of 0.94 and 0.73 for methylamine and formate, respectively, calculated in a Pearson correlation.

By including data on the wild-type state prior to an environmental or genetic perturbation, it becomes possible to distinguish which evolved changes were truly novel versus simply altering the acclimatized state. This allows physiological changes to be categorized into four patterns (Figure 1A): *restored*, *unrestored*, or *reinforced* refer to whether acclimatizing responses were reversed, left unchanged, or enhanced through evolution, whereas *novel* changes did not manifest during initial acclimation, but appeared only later during evolution. Importantly, these classifications can be applied to various levels of physiological processes – from alterations in gene expression, to protein activity, metabolite concentrations and flux, and even higher-order properties such as growth rate or fitness – and could conceivably differ between levels. Ultimately, this framework provides a “direction” to orient the interpretation of physiological changes that occurred during adaptation, revealing the level and degree to which adaptation either restores prior cellular states or finds novel solutions to improve growth or fitness. We hypothesized that physiological changes that are simply restorative would occur commonly, and would frequently arise in parallel between replicate evolved lineages. By sorting out these restorative changes, the novel and reinforcing changes that remain should more clearly reflect the physiological bases of adaptation. Particularly when these novel or reinforcing changes occur in parallel, they may identify loci in which the causative, beneficial mutations occurred.

As a model system in which to examine the interaction between acclimation and adaptation to perturbations, we employed a combination of metabolic engineering plus experimental evolution to study physiological and evolutionary responses to a novel, sub-optimal central metabolism in *Methylobacterium extorquens* AM1. As a facultative methylotroph, *M. extorquens* AM1 is capable of utilizing one-carbon (C_1_) compounds like methanol as a sole source of carbon and energy, as well as other multi-carbon compounds like succinate [13]. Its metabolism of C_1_ compounds is a complex process that requires over 100 different genes [14], many of which were acquired via horizontal gene transfer [15], [16]. C_1_ substrates such as methanol or methylamine are oxidized first to formaldehyde, and in wild-type (WT), this toxic intermediate is then oxidized to formate using a pathway linked to tetrahydromethanopterin (H_4_MPT), an analog of folate [15], [17] (Figure 1B). From formate, C_1_ units can be further oxidized into CO_2_ for the production of NADH, or assimilated into biomass [18], [19]. To create an engineered *Methylobacterium* (EM) strain, the native H_4_MPT-based pathway of formaldehyde oxidation was disabled and replaced by a functionally analogous, yet non-homologous C_1_ pathway. Two genetic changes were required to make EM: (1) the deletion of the *mptG* locus, which encodes the enzyme that drives the first committed step in H_4_MPT biosynthesis and is necessary for growth or survival in the presence of methanol [17], and (2) the introduction of an expression plasmid with two genes – *flhA* and *fghA*, both from *Paracoccus dentrificans* – that drive the oxidation of formaldehyde to formate using glutathione (GSH) as a C_1_ carrier [20]. The introduction of the engineered GSH-dependent pathway restores the ability of the Δ*mptG* strain to grow on methanol, however this EM strain is approximately 3-times slower growing than WT. Furthermore, the EM strain exhibits morphological abnormalities that arose from overexpression of the foreign GSH pathway [20].

Eight replicate populations (F1–F8) were founded from an EM ancestor and propagated on methanol for over 600 generations in batch culture to study adaptation to a novel metabolic module. Adaptation in the F populations was substantial, rapid, and largely methanol-specific [20]. The cellular abnormalities that emerged as a consequence of introducing the foreign pathway were also eliminated, representing an example of a restored (morphological) change. Several beneficial mutations have been identified in these evolved lines, including four from an isolate from the population with the highest fitness gains (F4). Notably, all four of these beneficial mutations altered gene expression. The targets and apparent physiological pressures acting upon these beneficial mutations are as follows. (1) Increased *pntAB* expression: switching from the native to the engineered pathway of formaldehyde oxidation eliminated the cell's only direct source of NADPH production, and a transhydrogenase encoded by *pntAB* can overcome this limitation by reducing NADP^+^ to NADPH using NADH [21]. (2) Increased *gshA* expression, which encodes an enzyme in GSH biosynthesis: GSH is needed to react with formaldehyde in the engineered pathway, and its recruitment into central metabolism might dilute GSH away from its native functions to protect against oxidative stress [22]. (3) Increased *icuAB*, which encodes a cobalt transporter: this mutation allowed cells to overcome metal limitation in the medium [10]. And (4), decreased expression of the introduced GSH pathway (i.e. *flhA* and *fghA*) [20], [23]: Foreign genes and plasmids introduced through engineering or natural gene transfers are often sub-optimal in terms of their sequence, expression, or activity for their new host and function [23]–[25]. Correspondingly, mutations that decreased expression of *flhA* and *fghA* balanced the benefits of formaldehyde oxidation with the costs of gene expression, and these occurred in all eight evolved populations through a variety of genetic mechanisms [23]. While these mutations to *pntAB*, *gshA, icuAB*, and the foreign pathway are known to have improved fitness in one or more lineages, many other changes in cellular physiology were also altered as a consequence. It is unclear whether these mutations produced novel or restorative physiological states, nor the extent to which these changes occurred in parallel across replicate populations.

Here, we sought to examine the extent to which evolution creates truly novel physiological states in the F lines, versus simply restoring acclimatizing processes towards WT-like levels. To this end, we used DNA microarrays to analyze changes in global gene expression from WT, to EM, to each of the eight F strains, and classified significant changes into patterns of restored, unrestored, reinforced, and novel gene expression as in Figure 1A. Without knowledge of acclimatizing processes, the substantial transcriptional changes observed in the evolved lineages would have been perceived as novel; however, our analysis revealed an overwhelming trend towards restoring gene expression to the WT state. Furthermore, whereas over 300 genes restored expression in parallel across all eight replicates, novel or reinforced changes tended to be unique to one or a few populations. Rare examples of parallelism amongst the novel or reinforced changes were particularly enriched for the loci with known beneficial mutations described above, or other probable candidates. One example of a highly parallel and beneficial reinforced change – PntAB transhydrogenase – translated into a restorative change in physiology, as it appeared to return NAD(P)(H) pools perturbed in EM back toward WT levels. Thus, incorporating information from physiological acclimation to a genetic or environmental perturbation can “orient” the interpretation of evolutionary adaptation, thereby distinguishing restorations from novelty, and greatly enriching for physiological changes that were causes, and not just consequences of increased fitness.

## Results

### Growth and fitness gains in the evolved strains were substantial and focused largely on formaldehyde oxidation

To interpret gene expression and other physiological data, we first characterized the growth rate and competitive fitness of WT, EM, and isolates of each F population after 600 generations of evolution. Relative growth rates measured using a high-throughput, automated robotic system [26]–[28] indicated the evolved isolates were now 1.95 to 2.5 times that of their EM ancestor on methanol, while WT was 3 times as fast (Figure 1C). Improvements in the F isolates were similar to the gains previously measured at the population level [20], suggesting that our isolates were representative of their respective evolved populations. Furthermore, these improvements in growth rate correlated well with increases in relative fitness, as determined by head-to-head competition [29], confirming that selection focused largely on exponential growth (Figure 1C).

Given that the sole difference between the slow EM strain and WT was the replacement of the formaldehyde oxidation pathway, we hypothesized that adaptation in the F lines would largely focus upon this stage of methanol metabolism. To test this hypothesis, we determined the specific growth rate of strains on two additional C_1_ substrates: methylamine, and formate. Growth on methylamine is nearly identical to growth on methanol, except that formaldehyde enters C_1_ metabolism by way of methylamine dehydrogenase [30]; while, in contrast, growth on formate skips the steps of formaldehyde oxidation altogether (Figure 1B). Relative to EM, the improvement of strains on methylamine was nearly comparable to their respective gains on methanol, while there were much smaller gains on formate (Figure 1D). The large difference between improvement in the selective environment (with methanol) versus formate, or succinate [20], contrasts with the generic improvements across substrates that was observed after adaptation of WT on methanol [29]. Overall, these data suggest that selection in the evolved lineages was focused predominantly focused on the formaldehyde oxidation pathway of C_1_ metabolism required for both methanol and methylamine growth.

### Evolved changes in global gene expression were mostly restorative and highly parallel across populations

**Figure 2:**
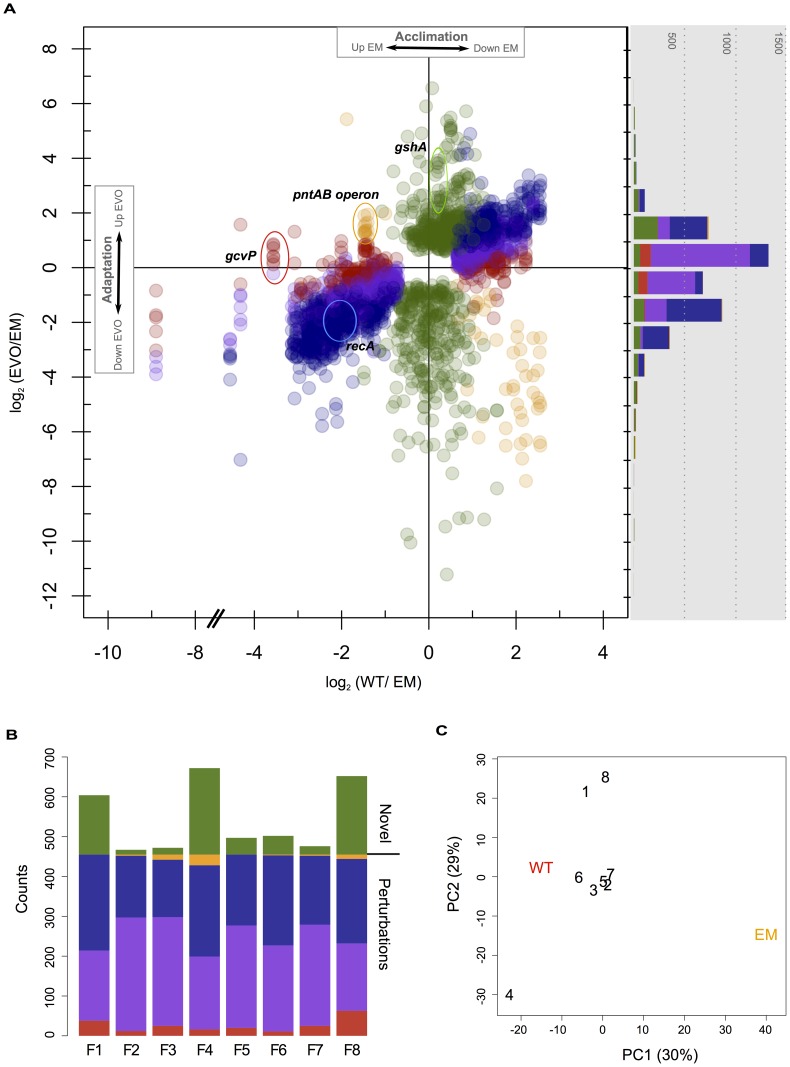
Microarray analysis of changes in gene expression in WT, EM, and each of the evolved strains. A) Genes with significant differences in expression in WT or the F isolates relative to EM. Each gene has a single value for the log_2_ difference in expression of WT relative to EM, and up to eight different values for each evolved strain. Changes in expression were categorized and colored as in Figure 1A as restored (blue), unrestored (red), reinforced (orange), or novel (green), as well as a fifth class of “partially restored” expression (purple). The histogram (right) bins each of these observations only considering adaptation, and thus just the differences between EM and the evolved strains. B) Instances of restored, unrestored, partially restored, reinforced, or novel expression across each of the eight evolved strains. C) Principal component analysis of all differentially expressed genes from physiological acclimation and adaptation.

To investigate large-scale changes in physiology arising due to the replacement (acclimation) and subsequent evolution (adaptation) of the formaldehyde oxidation pathway, we used DNA microarrays to examine differences in global gene expression. We identified 878 genes that were differentially expressed relative to EM: 455 of which arose as acclimatizing responses to metabolic engineering, while the remaining 423 genes appeared only in the evolved isolates. Patterns of restored, unrestored, reinforced, or novel gene expression were categorized by following the fate of EM perturbations (if present) into each of the evolved lineages. Due either to experimental noise or an intermediate reversal in gene expression, a significant number of genes fell in-between our criteria for restored and unrestored, and were thus classified as a fifth group of “partially restored” changes. We present changes in gene expression in two ways (Figure 2A). First is a scatter plot that depicts both the changes that occurred during acclimation to the introduced pathway (WT vs. EM, x axis), versus those that occurred during adaptive evolution (EVO vs. EM, y axis). Second, we present a histogram (grey box) that compiles these data solely in terms of the changes that occurred during adaptation.

The majority of gene expression changes that occurred in the evolved strains were not novel, but restored perturbations to a WT-like state. Genes whose expression was fully or partially restored greatly outnumber the other categories. This is apparent by the large number of restored and partially restored genes in the scatter plot (Figure 2A), as well as by tabulating the number of genes satisfying each category across independent evolved isolates (Figure 2B, blue and purple). The next most numerous category was novel changes, followed by unrestored and then reinforced. As an additional method to explore similarity between transcriptional profiles, we used principal component analysis (PCA). Including all significant expression changes, PC1 clearly separats EM from WT and the evolved isolates. In contrast, PC2 distinguishes three evolved isolates from the remainder: separating F4 from F1 and F8 (Figure 2C). This highlights that, despite the great degree of parallelism in restorative gene expression, the transcriptomes of a few F lines appear to be quite distinct. Considering just those genes that are perturbed in EM (i.e., acclimatizing responses, with no novel changes), all evolved isolates cleanly fall between EM and WT, while F4, F1, and F8 remain quite distinct (Figure S1A). Considering just novel changes, only F4 and the pair of F1 and F8 are distinct from the rest (Figure S1B).

**Figure 3:**
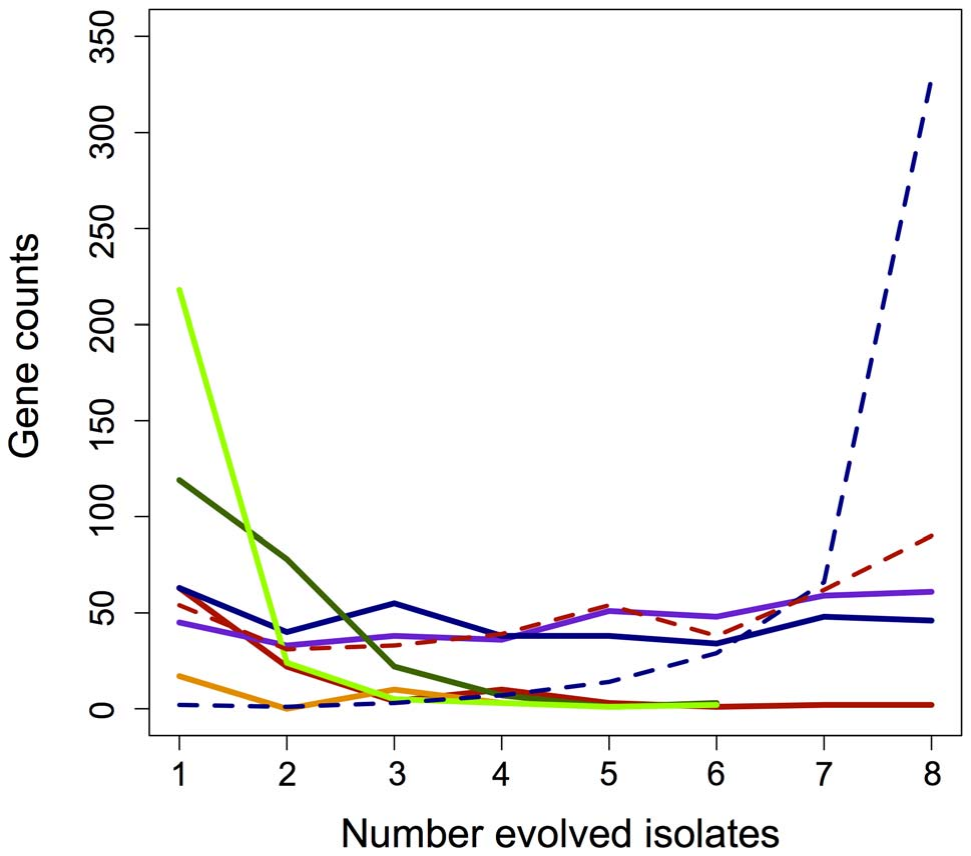
Parallelism of gene expression changes across categories. Instances of fully (blue) and partially (purple) restored, unrestored (red), reinforced (orange), or novel (green) changes in gene expression that occurred in parallel across the evolved lines. Novel is separated here into both increases (light green) and decreases (dark green) in expression for that gene. Dashed lines represent the parallelism of partially restored genes when combined with either fully restored (dashed blue) or fully unrestored (dashed red) changes.

Given that most of the 455 genes with perturbed expression in EM were restored during adaptation, we hypothesized that this class of changes may be particularly likely to occur in parallel across the F lines. For each gene with significant expression changes, we tabulated how many instances of each class occurred across the F lines (Figure 3). Only about 10% (46/455) of perturbed genes satisfied the strict criterion for restoration in all eight populations (solid blue). By grouping strictly restored genes with cases of partial restoration, 72% (328/455) perturbed genes were restored across all eight populations (dashed-blue), and 98% (444/455) moved toward WT in at least four populations. In contrast, partially restored changes had little affect when combined with fully unrestored genes. This tremendous degree of parallelism was not observed for novel expression changes. Over 70% (330/483) of these occurred in just one strain, and of those that occurred in two populations, 81% (83/102) of these were specific to the F1 and F8 isolates that exhibited a particularly distinct transcriptional pattern.

Gene expression that was restored or unrestored in perfect parallel highlights the major acclimatizing responses of EM to perturbations invoked by metabolic engineering. The 46 genes that were restored across all lineages function in heat shock and stress responses (including *recA*), C_1_ metabolism (components of methanol dehydrogenase), chemotaxis response regulators, and various genes putatively of phage origin. Conversely, only two genes – a glycine riboswitch (*META1_misc_RNA_19*) and a conserved hypothetical protein (*META2_0338*) were never restored in any lineage. In the adaptation of the F lines, we hypothesized that a greater number of restored genes would indicate a closer return to the wild-type state, and thus faster growth. This could occur either directly through mutations to pathways controlling the expression of perturbed genes, or secondarily as other physiological processes are restored (e.g., increase in growth rate or decrease in stress). Contrary to this hypothesis, however, growth rate did not correlate with either increased instances of restored or partially restored gene expression, or with a decrease in unrestored expression (Figure S2A–S2C). Furthermore, none of the four aforementioned loci known to have experienced beneficial mutations fell into this class. This suggests that the overall extent to which expression of evolved isolates returned to the WT-like state is not a good indicator of growth improvement.

### Instances of novel gene expression were unique to one or a few evolved strains

Many if not most evolution experiments focus only on changes that are novel with respect to their ancestor. Provided with only knowledge of the EM state and the commonly-used threshold of 2-fold differential expression, our analysis would have wrongly classified many instances of fully or partially restored and reinforced expression as being novel in the evolved lineages (see histogram in Figure 2A). However, by incorporating WT physiology, we were able to identify 423 genes whose expression is wholly novel in at least one evolved lineage. The number of genes with novel expression varied between the F strains from only 12 in F2, to 217 in F4 (Figure 2B). Most instances of novel expression were unique to one or a few evolved lineages, with the exception of a few loci. One might therefore expect that an increased number of novel changes would be correlated with higher fitness, however no correlation was found between growth rate and increased instances of novel gene expression (Figure S2D). In fact, the F1 and F8 strains share a large number of uniquely derived changes in gene expression - with functions in DNA transcription and translation, DNA synthesis, and a number of C_1_-related genes – yet are amongst the least improved lineages. In F4, many novel down-regulated genes (Figure 2A) are in fact instances of gene loss from a previously identified deletion on the *M. extorquens* AM1 megaplasmid [20], [31] that has been shown to be beneficial and recurring across experiments [32]. While individual cases of novel gene expression are no doubt important to growth and fitness gains in the F isolates, we found that these are in general less frequent than restorative changes, mostly restricted to one or a few strains, and on the whole a poor indicator of improvements gained in the F lines.

### Rare instances of reinforced gene expression highlight important links between acclimatizing and adaptive responses

The rarest, and perhaps most interesting class of gene expression changes were those that were reinforced, in which the acclimatizing response of EM to metabolic engineering was augmented through the evolutionary process. We identified only 30 genes with reinforced expression in at least one evolved isolate (7% of perturbed genes), which include the increased expression of the *pntAB* operon, the up-regulation of two genes with putative functions in cobalamin biosynthesis, the down-regulation of genes with predicted functions in fatty acid metabolism, and other genes with poorly-annotated functions that were down-regulated. Most genes with reinforced expression were unique to one or a few F strains, and remained unrestored or were restored in the other isolates (Figure 3). Unlike the above tests, instances of reinforced expression were strongly correlated with improvements in growth rate (Figure S2E; R^2^ = 0.87, p = 0.005), however the sample size of reinforced changes is small. As described above, *pntAB* was known to contain a beneficial mutation in its promoter in F4 [20], and these data now show that increased expression at this locus was not novel, but rather a response that arose first in the acclimation of EM and was reinforced through evolution.

We hypothesized that highly parallel instances of novel or reinforcing changes in gene expression might be enriched for loci with beneficial mutations. Although 306 genes showed parallel changes in expression across at least six populations, and 453 genes were either novel or reinforcing, only 5 instances were observed that satisfied both criteria, and they were all novel. We identifed those loci with known beneficial mutations (*gshA* and *icuAB*); one other gene with parallel increases (META1_0936, a putative type I secretion membrane fusion protein); and two genes with parallel decreases (META1_2657, a putative *soxC* sulfite oxidase; and META2_1007, a putative beta-lactamase). Regarding the parallelism of reinforcing changes, the three loci composing the *pntAB* operon were increased in 8/8 lineages relative to EM, but only significantly so in 4/8 lineages. This suggests that beneficial mutations may be particularly common in expression changes that are both parallel and buck the trend of restoration to the WT-like state.

### Divergent mechanisms lead to parallel increases in the expression and activity of PntAB transhydrogenase

**Figure 4:**
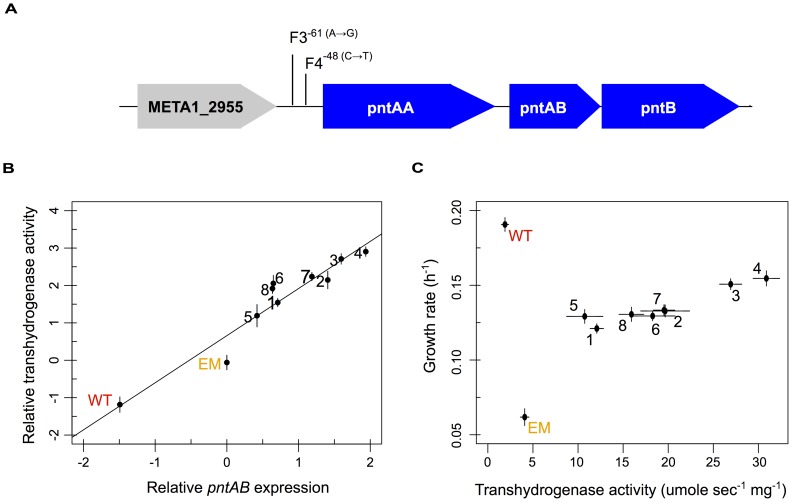
Multiple evolved mechanisms reinforce increased *pntAB* expression and transhydrogenase activity. A) Known (F4) and candidate (F3) mutations that increase *pntAB* expression. B) Relative increases in *pntAB* expression were highly correlated with increased overall transhydrogenase activity (Pearson correlation, r^2^ = 0.96 with p = 1.27×10^−5^). C) Plot of increasing transhydrogenase activity with increasing growth rate. WT is able to grow well in the absence of transhydrogenase; however, enzyme activity is significantly increased from WT to EM, and reinforced even further from EM to each of the evolved lineages (p<0.05, Welch two-sample *t*-test). A significant positive relationship exists between transhydrogenase activity and growth rate for the evolved isolates (Pearson correlation, r^2^ = 0.86 with p<0.01).

PntAB transhydrogenase functions in redox homeostasis, and thus it was intriguing to find that perturbed *pntAB* expression was not restored but actually increased further away from WT levels. We hypothesized that the consequence of increased *pntAB* could actually be restorative to the levels of pyridine nucleotides NAD(H) and NADP(H), despite its enhanced expression. We first examined the evolved isolates for mutations in the *pntAB* locus beyond that known for F4, and only one other strain – F3 – had a similar mutation in the upstream region (Figure 4A). Next, we investigated whether increased expression of *pntAB* equated to increased enzyme function. Transhydrogenase activity measured in WT, EM, and each of the evolved isolates closely mirrored changes in the expression of *pntAB* measured in the microarray analysis (Figure 4B). Our data suggest that, outside of F3 and F4, increased transhydrogenase in the F strains occurs either as a consequence of outside physiological changes (e.g., via allostery) or through *trans*-acting factors that drive increased expression in these lineages. We further examined the relationship between transhydrogenase levels and growth rate, and found a highly correlated positive relationship amongst the evolved F isolates (Figure 4C).

### The reinforcement of PntAB restores perturbations in NAD(P)(H) to wild-type

To determine whether the effect of increased transhydrogenase activity in the evolved strains was to restore the redox balance of pyridine nucleotides, we examined strain differences in the ratios of NADPH/NADP^+^ and NADH/NAD^+^. Interpreting changes in the steady-state concentrations of metabolites such as NADPH is complicated by the fact that these values represent a balance between production (such as by transhydrogenase) and consumption via biosynthesis. As there is relatively little degeneracy in the network of biosynthetic reactions, the rate of NADPH use should be nearly directly proportional to growth rate, such that mutations that increase the cell's capacity to grow can actually decrease the steady-state concentration of currency metabolites. Indeed, data from a variety of other organisms, such as *Escherichia coli* [33] and *Lactococcus lactis* [34] grown at different rates in chemostats have confirmed this intuition.

**Figure 5:**
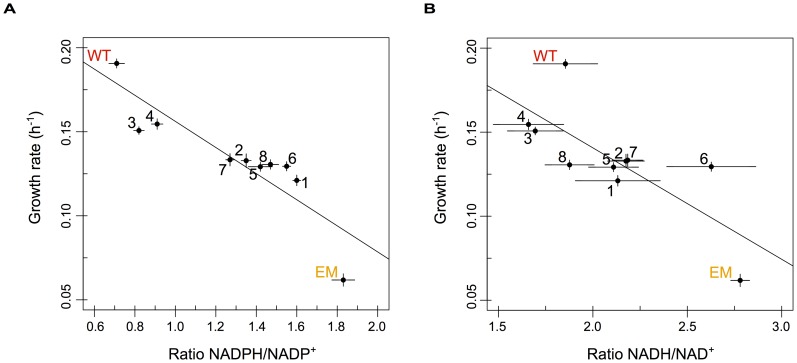
The redox states of pyridine nucleotides are perturbed in EM but restored through evolution. The relative ratios of NADPH/NADP^+^ (A) and NADH/NAD^+^ (B) plotted against growth rate for WT, EM, and each of the evolved strains. The redox states of NADP(H) and NAD(H) were perturbed in EM but returned toward WT-like values in almost all of the evolved lineages. Ratios were highly correlated with growth rate for both NADP(H) (r^2^ = −0.88 with p = 6.9×10^−4^) and NAD(H) (r^2^ = −0.76 with p = 0.011) in a Pearson correlation.

Consistent with the above expectations, the slow-growing EM strain possessed a much higher ratio of both NADPH/NADP^+^ and NADH/NAD^+^ than WT. Including the evolved isolates, the ratios (or redox state) of reduced to oxidized NADP(H) and NAD(H) were both highly negatively correlated with growth rate, such that faster-growing strains possess substantially lower ratios for each (Figure 5A and 5B, respectively). Variation in levels of NADPH/NADP^+^ also correlated well with changes in *pntAB* expression: strains with significant increases in *pntAB* (n = 4 strains) showed significantly lower NADPH/NADP^+^ ratios than those with marginal increases (p<0.05, Welch two-sample *t*-test); however, the same was not true for NADH/NAD^+^ ratios. Even amongst strains with significantly increased *pntAB* expression, those with *cis*-acting mutations (F3 and F4) were significantly faster and had lower NADPH/NADP^+^ ratios than strains with significant increases apparently driven in *trans* (F2 and F7). Importantly, almost all strains statistically significantly restore the redox states of NADP(H) and NAD(H) towards WT-like levels. Overall, these data suggest that the reinforcement of transhydrogenase activity increased the rate of NADPH production and drove the restoration of pyridine nucleotide metabolism back toward a WT-like state through an apparent variety of adaptive mechanisms.

## Discussion

Organisms are constantly pressured by ever-changing and potentially disruptive cellular and environmental conditions. Large-scale changes in physiology can occur due to ecological or environmental transitions, or upon sudden changes in genomic composition due to mutation, horizontal gene transfer, or genetic engineering in the laboratory. When a perturbed, sub-optimal physiology persists over multiple generations, transient acclimatizing responses begin to overlap with responses from evolutionary adaptation. Conceptually, processes of physiological acclimation and adaptation are intimately linked: as beneficial mutations should revert many acclimatizing processes from a perturbed to a baseline physiological state. In practice, the interplay between acclimatizing and adaptive responses to perturbations has often been ignored, leading to the scenario where large-scale, parallel restoration of physiology to a pre-stress state will appear as novel. We argue that a proper interpretation of evolved physiological states is only possible given knowledge of the initial acclimation to a new environment or genomic composition.

Our work sought to determine the extent to which cells adopt novel versus restorative physiological states by examining acclimatizing and adaptive responses to a novel central metabolism. We utilized a strain of *M. extorquens* AM1 (EM) that was metabolically engineered to utilize a foreign, GSH-based central pathway to oxidize formaldehyde during growth on C_1_ compounds, and was subsequently propagated in eight replicate F populations for over 600 generations of evolution to optimize growth using the engineered pathway. The physiology of the EM ancestor was perturbed in many ways: it was three-fold slower; adopted an elongated, curved or branched cell morphology [20], and exhibited a unique density-threshold for growth on methanol [35]. Here we document two additional levels of physiological perturbation: microarray analyses revealed 455 genes with altered expression from WT to EM, as well as perturbations in the central redox cofactors, NAD(H) and NADP(H). By orienting our analyses based upon the initial acclimation from WT to EM, we categorized evolved changes as restored, unrestored, reinforced, or novel as in Figure 1A. Given the particularly interesting connection between acclimatizing and adaptive processes in reinforcement, we further examined the systems-level consequences of enhanced PntAB activity.

The major pattern seen for evolved changes in physiology was an overwhelming trend to return to a wild-type state. Our work highlights a few general trends to be explored in other systems. First, the majority of gene expression differences distinguishing the ancestor and the evolved isolates were not novel, but instead restorative. Most restored genes were not themselves targets of beneficial mutations, but altered in response to other changes such things as NAD(P)(H) levels, or indirectly, improved growth rate (increased methanol dehydrogenase), or reduced stress (decreased *recA* and heat shock proteins). So much of gene expression was restorative that it outweighed instances of novel expression in all evolved strains. Similarly, PCA analysis confirmed that expression in the evolved isolates was more like WT than their common EM ancestor. One interesting future direction would be to examine the temporal component of adaptation, studying the degree to which physiology is restored as populations acquire sequential beneficial mutations. Second, the restoration of WT physiology occurred highly in parallel. Indeed, the vast majority of genes were restored, at least partially so, in all eight lineages. This is perhaps intuitive as a shared set of acclimatizing processes from EM were simply “turned off” in the case of stress-related responses, or “turned up” in the case of growth related genes, in the evolved lines. Without specific knowledge of these acclimatizing processes, however, most of these restorative changes would be wrongly classified as novel (Figure 2A, histogram). Third, some acclimatizing processes were left unrestored because physiological adaptation cannot, or has not yet, addressed these perturbations. These may represent fundamental and perhaps inescapable differences separating WT and EM physiologies. And finally, changes that are both highly parallel and either novel or reinforced are potentially enriched for loci targeted by beneficial mutations, and thus causal changes during adaptation. Increases in expression in *gshA* (6/8 novel), *icuAB* (6/8 novel), and *pntAB* (4/8 reinforced) are all outliers when comparing the parallelism of changes in each category across genes (Figure 3). In fact, by filtering out (highly parallel) restorative changes, we find only 19 genes (out of 878) that are novel or reinforced changes in half or more of the evolved strains. Including the parallel, beneficial decreases in the expression and/or activity of the foreign pathway that occurred in 8/8 strains [23], parallel changes in gene expression that are not restorative appear to be particularly enriched for beneficial mutations that drove adaptation.

Looking closer, we did find variation in how the various F lines adapted to an engineered C_1_ metabolism. Novel expression of genes very rarely occurred in more than one strain, and where observed, it was nearly always to the F1 and F8 strains. These isolates consistently showed different transcriptional profiles than the other F isolates, not only amongst novel genes, but also in the number, types, and degree to which genes are restored. Interestingly, both F1 and F8 are also amongst the slowest growing of the F strains, suggesting perhaps the presence of a multi-peaked fitness landscape in which these strains have found a local optimum. While it appears that the F populations restored many genes in parallel, and share at least a few common molecular and physiological mechanisms, additional work is needed to understand the full extent to which these strains found parallel versus divergent paths to optimize growth using an engineered central metabolism.

Reinforcing changes to physiology, while rare in our system, are an important link between processes of physiological acclimation and adaptation. We focused on one particular instance of reinforcement – the up-regulation of *pntAB* transhydrogenase – to investigate both the genetic basis for enhancing expression beyond acclimation and to uncover its physiological consequences. Normally, *pntAB* is expressed during multi-C and not C_1_ growth [36], however in EM, the only direct source of NAD(P)(H) production was lost with the deletion of the native pathway of formaldehyde oxidation. This perturbation might invoke increased *pntAB* to maintain NAD(P)(H) homeostasis during growth on methanol. Supporting this hypothesis, the deletion of *pntAB* was found to be neutral for C_1_ growth in WT but lethal in the EM strain (H.-H. Chou, data not shown), and the mutation in the F4 lineage that drives increased *pntAB* expression provides a 10% selective benefit in the ancestral background [20]. These results demonstrate the irreplaceable role of *pntAB* as an acclimatizing response in EM, and the benefit of reinforcing this function even further through adaptation.

While the increased expression and activity of PntAB transhydrogenase was reinforcing, this translated into a restorative effect upon metabolism. All eight F strains increased transhydrogenase activity significantly, despite significant increases in expression for only half of these. Upon sequencing the genomic neighborhood of *pntAB*, we identified only two strains – F4 and F3 – that possess known or candidate mutations to drive increased expression. As for the physiological consequence of increased transhydrogenase activity, all evolved strains tend to restore NAD(P)(H) metabolism, and strains with greater increases to *pntAB* – particularly the pair with mutations in the upstream region – have levels of NAD(H) and NADP(H) that are the closest to WT. The reinforcement of *pntAB* expression and transhydrogenase activity, as well as the restoration of NAD(P)(H) levels, are both well correlated with increased growth rate in the F populations. By increasing activity during acclimation, and reinforcing this response further during adaptation, transhydrogenase activity appears to have been critical in maintaining and improving growth in the EM strain.

Information on acclimatizing and adaptive responses in the engineering and evolution of EM allowed us to develop a framework in which to examine the true nature of evolved physiological change. We defined four basic patterns to describe not only novel changes to physiology, but also changes that restore, disregard, or reinforce the initial acclimatizing responses to perturbations (Figure 1A). To our knowledge, this linkage between immediate physiological acclimation and subsequent adaptation has explicitly been explored only once before [12]. This paper described a large number of “compensatory” changes in gene expression that effectively restored the wild-type (glucose-grown) state during the acclimation and experimental evolution of *E. coli* to sub-optimal carbon sources. The commonalities between adaptation to a poor environment versus a novel, suboptimal metabolic pathway are remarkable: whereas their study showed that 87% of genes were restored after adaptation to a poor substrate, we found that on average that 93% of genes were restored after adaptation to the foreign pathway; their change was environmental, ours genetic. Furthermore, reinforcing changes are reminiscent of the fixation of traits via genetic accommodation or assimilation [37]–[39], in that both processes stem from exposure to genetic or environmental stressors to reveal beneficial phenotypes that are “assimilated” and possibly reinforced by positive selection. However, in genetic assimilation, stress-induced phenotypes arise from cryptic genetic variation in populations [40] while, at least for the reinforced up-regulation of *pntAB* expression, the initial response required no standing genetic variation at all. In fact, the initial acclimatizing response of EM to increase *pntAB* was merely a generic response to NADPH shortage typically experienced during growth on multi-carbon substrates such as succinate [36], that was co-opted for methanol growth in EM, and further increased and optimized by selection during adaption of the F lines.

Overall, our results suggest that much of evolutionary adaptation effectively relieves processes of physiological acclimation. Rather than “reinvent the wheel” of C_1_ metabolism, a few causal mutations in the adaptation of the F populations propagated through physiology to restore WT homeostasis. In fact, more changes in gene expression occurred as a result of acclimation to genetic engineering (n = 455) than novel changes seen in any of the isolates after 600 generations of experimental evolution (12 to 217). Beneficial mutations were enriched toward novel and reinforcing changes that occurred in parallel. By distinguishing acclimatizing versus adaptive processes, a more accurate depiction on the nature and parallelism of physiological evolution is revealed.

## Materials and Methods

### Growth conditions

All growth was performed using a modified “Hypho” minimal medium as in [20]. One liter of Hypho was prepared from 799 mL of deionized water, 100 mL phosphate salts (25.3 g of K_2_HPO_4_ plus 22.5 g NaH_2_PO_4_ in 1 L deionized water), 100 mL sulfate salts (5 g of (NH_4_)_2_SO_4_ plus 0.98 g MgSO_4_ in 1 L deionized water), and 1 mL of modified, high-iron “Vishniac” trace metal solution [10], [20]. All solutions were autoclaved separately and combined under sterile conditions, and the final medium was stored in the dark. Carbon substrates added just prior to inoculation consisted of: 20 mM methanol, 3.5 mM sodium succinate, 15 mM methylamine hydrochloride, or 20 mM sodium formate. Growth experiments were initiated by inoculating 10 µL of freezer stock into 9.6 mL Hypho in a 50 mL Erlenmeyer flask containing 10 mM methanol and 1.75 mM succinate plus 50 µg/mL kanamycin. Flasks were grown at 225 rpm in a 30°C shaker-incubator until reaching stationary phase (2–4 days). A second acclimation cycle was accomplished by transferring 150 µL of saturated culture into 9.45 mL fresh medium with 0.5× kanamycin plus the carbon substrate to be tested; then transferred again into the same conditions for experimental (measured) growth. A 1∶64 dilution of cultures with the given substrate concentrations allowed for six doublings per growth cycle.

All physiological assays (e.g., microarray analyses, enzyme assays, metabolite concentrations) were performed using cells that had reached half-maximal density following transfer from acclimation cultures also grown on methanol. This protocol results in eleven doublings of growth in a consistent environment while ensuring cells were still growing exponentially at the time of harvest. This gave the maximal possible time to approach steady-state physiology while staying within the constraints of the selective conditions. Furthermore, since it was previously found that the EM ancestor exhibits a unique cell-density threshold for growth [35], it would not have been possible to have diluted the cultures much more than the 1/64 used here.

Specific growth rates were determined in 48-well plates using a high-throughput, robotic system that automates measurements of optical density (i.e., OD_600_) in growing cultures at timed intervals [27]. This system consists of a plate-shaking tower, a plate reader, a robotic arm, and de-lidding station that transfer cultures between growth and measurements, all of which is scheduled with an open software manager program [26]. Strains for growth measurements were inoculated first into flasks, transferred to plates with for an acclimation phase, and transferred once more for measurement during the third cycle. All growth was performed in 640 µL total medium and were transferred in a 1/64 dilution (10 µL culture into 630 µL medium). To limit clumping and reduce noise in OD_600_ measurements in growing cultures, 0.1 mg/mL of prepared cellulase enzyme (Sigma-Aldrich, St. Louis, MO) was added to the growth medium (SMC, unpublished). The specific growth rate was calculated from the log-linear phase of growth for at least triplicate cultures of each strain using an open software analysis package [28].

### Strain construction and evolution

Strains and plasmids relevant to this study are listed in Table S1 and were generated previously, unless otherwise noted. The ancestral strains for the F populations were described previously [20]. Briefly, they derive from two WT *M. extorquens* AM1 strains - one that is naturally pink (CM501), and another that is white (CM502) due to a neutral mutation in carotenoid biosynthesis [41] – to limit contamination between cultures. The EM strain was constructed in two steps: 1) the H_4_MPT-dependent pathway was disabled by deleting the *mptG* locus (encoding β-ribofuranosylaminobenzene 5′-phosphate synthase), the product of which drives the first committed step in the H_4_MPT biosynthesis [42]; and 2) the introduction of a GSH-dependent formaldehyde oxidation pathway on the plasmid pCM410 – which expresses the genes *flhA* (encoding *S*-hydroxymethyl-GSH dehydrogenase) and *fghA* (encoding *S*-formyl-GSH hydrolase) from *Paracoccus denitrificans* –into the Δ*mptG* backgrounds, generating completed pink (CM701) and white (CM702) EM strains [20].

Eight replicate populations were founded from either the pink (odd populations; CM701) or white (even populations; CM702) EM strains and evolved for over 600 generations in 9.6 mL Hypho medium plus 15 mM methanol in batch culture with transfers of 1/64 of the volume every four days for the first 300 generations, and every two days thereafter. These evolved “F” populations (F1-8) were streaked at generation 600 onto Hypho agar plates to isolate colonies for further characterization. In addition to the previously characterized isolate from the F4 population, CM1145 [20], we chose for this study the second of three random isolates from each of the other F populations for further investigation (Table S1).

Other strains relevant to this study were as follows. Fluorescence-based fitness assays required an EM reference strain (CM1232) that had been generated by replacing the *katA* locus with *mCherry* driven by a constitutive *P_tac_* promoter [20]. To standardize the use of kanamycin in all cultures, we used a WT strain in which the *kan* resistance marker was inserted into *katA* (CM611) [29].

### Competition assays of relative fitness

The relative fitness of WT and evolved strains was assessed in a head-to-head competition of co-cultures with a fluorescently-labeled reference as in [29]. Briefly, fully-grown cultures of WT and each evolved isolate were mixed in roughly equal optical densities with an EM strain expressing *mCherry* (CM1232). A sample of this mixture (T_0_) was diluted with Hypho plus 8% DMSO and stored at −80°C in 96-well plates; the rest was diluted 1∶64 into 640 µL of Hypho methanol medium in a 48-well plate and incubated with shaking at 30°C for 4 days, after which samples of the co-culture after competition (T_1_) were frozen for later analysis using flow cytometry. Because of the 4-day growth cycle, this amortizes fitness over all growth phases (i.e., lag, exponential, and stationary).

The ratio of labeled to unlabeled cells before and after co-culture growths was measured using a BD LSR Fortessa flow cytometer with an HTS attachment for 96-well plates (BD Biosciences, San Jose, CA). Recently it was found that the forward scatter (FSC) and side scatter (SSC) settings used in earlier work [20] systematically underestimated fitness increases relative to EM because of the cells' larger size. Here we set both scatter measurements set to 300 V to accommodate small bacterial cell sizes [23], and the flow-rate was adjusted to the lowest setting to produce reliable measurements of labeled and unlabeled events in dilute co-cultures. The ratio of nonfluorescent to fluorescent cells before (R_0_, from T_0_) and after (R_1_, from T_1_) competition were used to calculate the fitness (W) of strains relative to the EM reference (CM1232) using the following formula, assuming a 64-fold expansion of cells from six doublings per growth cycle:
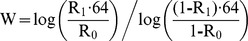



### Microarray analyses

Triplicate cultures of strains were grown to half-maximal OD_600_ in 15 mM methanol before harvesting and total RNA extraction using the RNeasy kit (Qiagen, Valencia, CA). Genomic DNA was removed using the TURBO DNA-free kit (Ambion, Austin, TX) and the RNA samples were concentrated using Amicon Ultra centrifugal filters (Millipore, Billerica, MA). Microarray analyses for all (n = 30) samples were performed by MOgene, Inc (St. Louis, MO) using one-color cDNA labeling and hybridization. The array probes and platform were designed previously [43] to include 60-mer oligonucleotides that provide two or more probes for confirmed and predicted ORFs in the *Methylobacterium* genome [31]. Raw and normalized expression data are available from the Gene Expression Omnibus, accession GSE42116. Pre-processing, normalization, and analysis of expression data was performed using the limma package [44], [45] with Bioconductor [46] and R [47]. Differentially expressed genes were identified by the proportion of differentially expressed probes in a limma contrast given: 1) at least two-thirds probes significant at p<0.05 in the moderated *t*-statistic, 2) at least one-half of probes significant at p<0.01, and 3) all significant probes with uniform changes either up or down. Probes that met these criteria were averaged in each strain to estimate the log_2_ difference in expression relative to EM. Genes differentially expressed in both EVO:EM and EVO:WT contrasts, and in the same direction, were classified as novel. Expression perturbations from acclimation were identified in a WT:EM contrast and further partitioned given information from EVO:EM and EVO:WT contrasts to define patterns of: restored expression, given an EVO:EM change back in the direction of WT expression; unrestored expression, given no EVO:EM difference but a significant EVO:WT difference; and reinforced expression, having an EVO:EM difference in the same direction (up or down) as the change from WT to EM (Figure 1A). Partially restored genes showed no EVO:EM difference and were not significant in a EVO:WT contrast. Principal component analysis was used to cluster and contrast the expression profile of WT, EM, and evolved strains, and was calculated using the *prcomp* function in R with scaling to account for large variance of expression changes between genes.

### Transhydrogenase enzyme activity

Cultures for the determination of TH activity and NAD(P)(H) ratios (below) were grown to half-maximal OD_600_ on methanol, spiked with another 15 mM methanol, and allowed to return to mid-exponential growth for approximately 16 hours to increase yield. Cultures for transhydrogenase activity measurements were pelleted and washed with 50 mM Tris-HCl (pH 7.5) before storage at −80°C. Upon thawing, cells were re-suspended in 2 mL Tris buffer and lysed by bead beating (MP Biomedicals, Solon, OH). Cell extracts were centrifuged for less than 15 s to collect the beads. The supernatant was removed and combined with a reaction mix consisting of: 20 µL of 40 mM MgCl_2_ (10×), 20 µL 5 mM NADPH (10×), 20 µL 10 mM 3-acetylpyridine adenine dinucleotide (10×), plus Tris buffer to equal 200 µL, total, in a 96-well plate. The increase in absorbance at 375 nm was measured immediately after addition of the reaction mix and the slope of the linear regression was used to calculate transhydrogenase activity (µmole of 3-acetylpyridine adenine dinucleotide reduced sec^−1^ mg^−1^) as follows: TH activity (µmole sec^−1^ mg^−1^) = slope (sec^−1^)×1/exctinction coefficient (0.0051 mol cm L^−1^)×1/path length (0.42 cm^−1^)×reaction volume (0.2 mL)×1/cell protein (mg)×1000 (conversion to µmole L^−1^).

### Measurement of relative NAD(H) and NADP(H) concentrations

Cell extracts for the measurement of pyridine nucleotide concentrations were prepared as follows. Metabolism in mid-exponential cells was quenched using vacuum-filtration and rapid immersion into hot extraction solutions. Oxidized pyridine nucleotides (NAD^+^ and NADP^+^) were selectively preserved in an acidic extraction solution consisting of 100 mM HCl plus 500 mM NaCl; reduced species (NADH and NADPH) were extracted using a basic solution of 100 mM NaOH plus 500 mM NaCl. For both acidic and basic extractions, 750 µL of culture was vacuum-filtered onto 0.45 µm nylon membranes (Millipore, Billerica, MA), immediately immersed into the appropriate extraction solution, briefly vortexed, and heated to 95°C for 5 m. Extracts were again briefly vortexed, centrifuged at maximum speed for 30 s, and the supernatant removed, flash frozen, and stored at −80°C for later use. Three biological replicates stemming from separate inoculations were extracted for each strain.

Pyridine nucleotides in cell extracts were quantified using enzymatic cycling [48] with alcohol dehydrogenase (ADH) or glucose-6-phosphate dehydrogenase (G6PDH) to measure NAD(H) and NADP(H), respectively. Each assay was performed using 20 µL of either acidic extraction solutions for oxidized species, basic solutions for reduced, or a serial dilution of (reduced) standards. For NAD(H), to 20 µL of cell extract or standard was added 180 µL of master solution consisting of: 20 µL 1 M bicine (pH 8.0) plus 40 mM EDTA (10×), 20 µL of 16.6 mM phenazine ethosulfate (10×), 20 µL of 4.2 mM thiazolyl blue tetrazolium bromide (10×), 20 µL of 100% ethanol, 2 µL of ADH (Sigma-Aldrich, St. Louis, MO) at 0.1 U/µL, and 98 µL water. The same mixes were used for the determination of NADP(H) with G6PDH, except that ethanol and ADH were replaced by 20 µL of 50 mM glucose-6-phosphate (10×) and 2 µL of G6PDH at 0.1 U/µL. Assays were conducted in 96-well plate format and measured in a Safire2 spectrophotometer (Tecan, Morrisville, NC) at 30°C by following the increase in absorbance at 550 nm over time.

## Supporting Information

Figure S1 Principal component analysis of novel (A) and perturbed (B) gene expression. Note that F1 and F8 are quite unique and cluster for both categories of genes. F4 is only particularly distinct for novel genes, largely due to the many decreases caused by the large deletion on the megaplasmid.
(TIF)

Figure S2 Correlations of growth rate with the number or percent of genes in each class. Growth rate on methanol versus the percent of restored (A), partially restored (B), or unrestored (C) perturbations; or the number of genes with novel (D) or reinforced (E) expression.
(TIF)

Table S1 Strains and plasmids relevant to this study.
(DOCX)
