# Supplementary figures and images for: Evolution after Introduction of a Novel Metabolic Pathway Consistently Leads to Restoration of Wild-Type Physiology
Source: PLoS Genet. 2013 Apr 4;9(4):e1003427. doi: 10.1371/journal.pgen.1003427 (PMC3616920; doi:10.1371/journal.pgen.1003427)

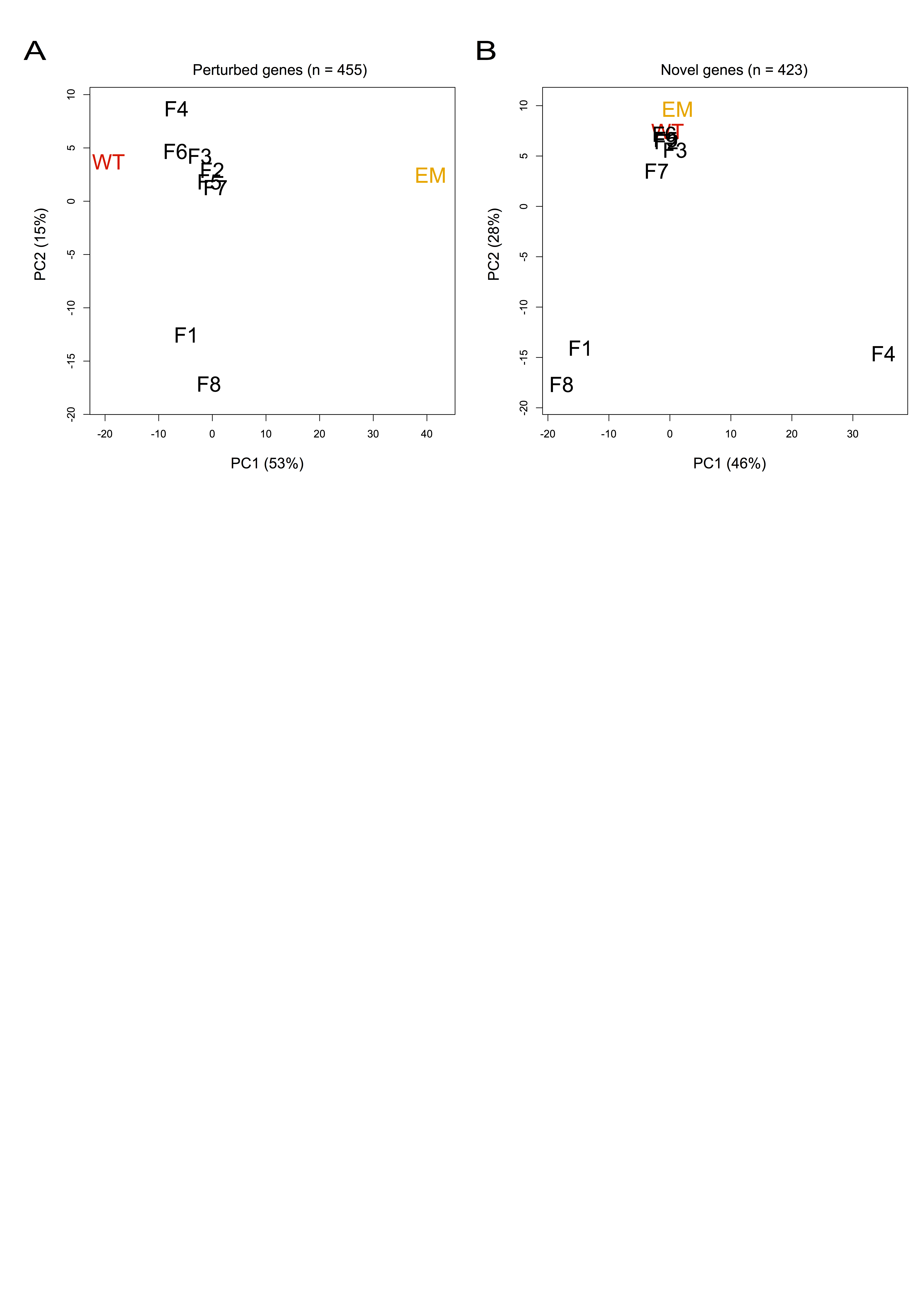

Supplement: Figure S1 — Principal component analysis of novel (A) and perturbed (B) gene expression. Note that F1 and F8 are quite unique and cluster for both categories of genes. F4 is only particularly distinct for novel genes, largely due to the many decreases caused by the large deletion on the megaplasmid. (TIF) [file pgen.1003427.s001.tif]

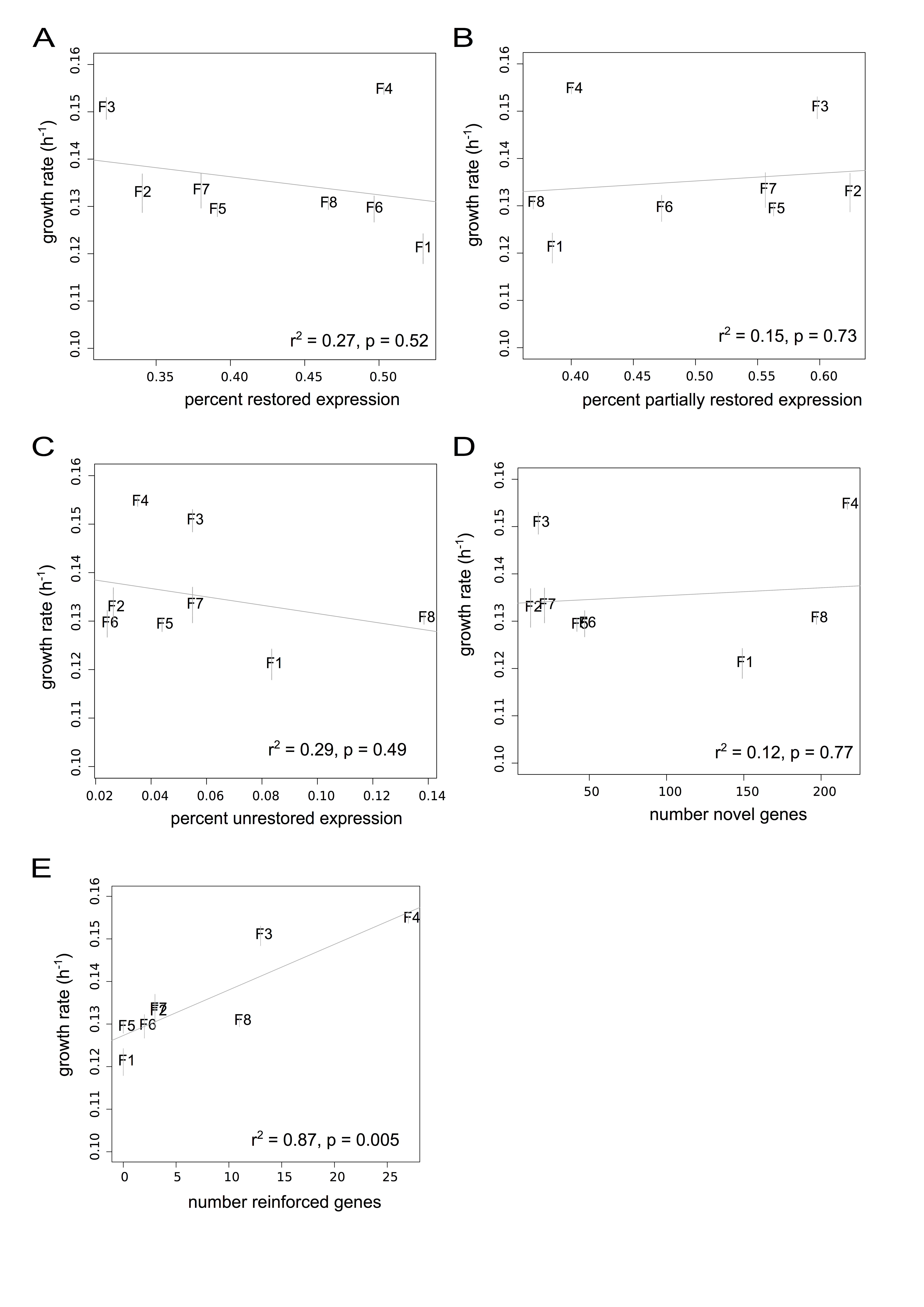

Supplement: Figure S2 — Correlations of growth rate with the number or percent of genes in each class. Growth rate on methanol versus the percent of restored (A), partially restored (B), or unrestored (C) perturbations; or the number of genes with novel (D) or reinforced (E) expression. (TIF) [file pgen.1003427.s002.tif]
