# Supplementary material for: Evolution after Introduction of a Novel Metabolic Pathway Consistently Leads to Restoration of Wild-Type Physiology
Source: PLoS Genet. 2013 Apr 4;9(4):e1003427. doi: 10.1371/journal.pgen.1003427 (PMC3616920; doi:10.1371/journal.pgen.1003427)
Supplement: Table S1 — Strains and plasmids relevant to this study. (DOCX) [file pgen.1003427.s003.docx]

**Table S1 – Strains and plasmids relevant to this study.**

| **Strain or plasmid** | **Description** | **Source** |
| --- | --- | --- |
| **Strains** |  |  |
| CM501 | Pink-colored WT *Methylobacterium extorquens* AM1 | [1] |
| CM502 | White-colored WT, *crtI*^502^ | [1] |
| CM508 | Δ*mptG* | [1] |
| CM611 | *crtI*^502^, *katA::kan* | [2] |
| CM624 | *crtI*^502^, Δ*mptG* | [3] |
| CM701 | Δ*mptG*, pCM410; pink engineered *M*ethylobacterium (EM) | [3] |
| CM702 | *crtI*^502^, Δ*mptG*, pCM410; white EM strain | [3] |
| CM1232 | *crtI*502, *katA::loxP-t_rrnB_-P_tacA_-mCherry-t_T7_*, Δ*mptG*, pCM410 | [3] |
| CM1727 | Evolved isolate from F1 population at generation 600 | This study |
| CM1730 | Evolved isolate from F2 population at generation 600 | This study |
| CM1139 | Evolved isolate from F3 population at generation 600 | This study |
| CM1145 | Evolved isolate from F4 population at generation 600 | [3] |
| CM1739 | Evolved isolate from F5 population at generation 600 | This study |
| CM1742 | Evolved isolate from F6 population at generation 600 | This study |
| CM1745 | Evolved isolate from F7 population at generation 600 | This study |
| CM1748 | Evolved isolate from F8 population at generation 600 | This study |
|  |  |  |
| **Plasmids** |  |  |
| pCM410 | *P_mxaF_-flhA-fghA*; Km^r^; GenBank acession FJ389188 | [3] |

**References**

1. Marx CJ (2008) Development of a broad-host-range *sacB*-based vector for unmarked allelic exchange. BMC Res Notes 1: 1. doi:10.1186/1756-0500-1-1.

2. Lee M-C, Chou H-H, Marx CJ (2009) Asymmetric, bimodal trade-offs during adaptation of *Methylobacterium* to distinct growth substrates. Evolution 63: 2816–2830. doi:10.1111/j.1558-5646.2009.00757.x.

3. Chou H-H, Chiu H-C, Delaney NF, Segrè D, Marx CJ (2011) Diminishing returns epistasis among beneficial mutations decelerates adaptation. Science 332: 1190–1192. doi:10.1126/science.1203799.
